# Contrasting groups’ standard setting for consequences analysis in validity studies: reporting considerations

**DOI:** 10.1186/s41077-018-0064-7

**Published:** 2018-03-09

**Authors:** Morten Jørgensen, Lars Konge, Yousif Subhi

**Affiliations:** 1 Faculty of Health and Medical Sciences, University of Copenhagen, Copenhagen, Denmark; 2 Copenhagen Academy for Medical Education and Simulation, Capital Region of Denmark, Copenhagen, Denmark; 3 Department of Ophthalmology, Zealand University Hospital, Vestermarksvej 23, DK-4000 Roskilde, Denmark

**Keywords:** Medical education, Messick’s validity framework, Contrasting groups, Standard setting, False positives, False negatives

## Abstract

**Background:**

The contrasting groups’ standard setting method is commonly used for consequences analysis in validity studies for performance in medicine and surgery. The method identifies a pass/fail cut-off score, from which it is possible to determine false positives and false negatives based on observed numbers in each group. Since groups in validity studies are often small, e.g., due to a limited number of experts, these analyses are sensitive to outliers on the normal distribution curve.

**Methods:**

We propose that these shortcomings can be addressed in a simple manner using the cumulative distribution function.

**Results:**

We demonstrate considerable absolute differences between the observed false positives/negatives and the theoretical false positives/negatives. In addition, several important examples are given.

**Conclusions:**

We propose that a better reporting strategy is to report theoretical false positives and false negatives together with the observed false positives and negatives, and we have developed an Excel sheet to facilitate such calculations.

**Trial registration:**

Not relevant.

**Electronic supplementary material:**

The online version of this article (10.1186/s41077-018-0064-7) contains supplementary material, which is available to authorized users.

## Background

Historically, surgery was learned by practicing on patients. To some extent, this is still the practice today. Simulation training enables practice and learning on a simulator before treating patients. Pushing the trainees up the learning curve before operating on patients leads to better outcomes [1]. There is an increasing number of simulators available today to facilitate such a process. However, validity evidence behind well-intentioned simulation training programs and interventions are crucial to ensure training that is relevant to clinical practice. Increasingly, efforts are made to provide such validity evidence: number of publications with validity assessments of surgical simulators have increased to 70–90 studies/year in years 2014–2016 from approximately 30 studies/year in years 2008–2010 [2].

In the contemporary validity framework by Samuel Messick, validity of a construct is explored from five sources [3]. One such source is the consequential validity, which explores the potential and actual consequences of a defined standard or a test. In our systematic review of current trends in validity studies, we found that consequential validity was explored the least of the five sources [2]. Based on such findings [4, 5], we think that attention needs to be given to consequential validity to facilitate its use.

Surgical education is moving away from time-dependent learning to competency-based learning. Competency-based learning ensures certification of the trainees with satisfactory levels of performance, skills, and knowledge [6]. An important issue in competency-based learning is the establishment of a competency standard that discriminates the trainees based on a defined level of competence. A standard is a score or a level of competency needed for a particular purpose and a standard can be a score needed to pass a test [7].

There are several methods to set such standards. The standard for when a certain level of expertise is reached is set by identifying cut-off points on different measures of performance, which can be rating scores or simulator metrics. One approach is the contrasting groups’ method that is a participant-based method where performance of a certain procedure is evaluated between participants of different expertise levels, e.g., novices and experts. In a study aimed at setting pass scores for surgical tasks using Objective Structured Assessment of Technical Skill, Montbrun et al. demonstrated that contrasting groups identify cut-off points at levels that are similar to those identified using other methods (i.e., borderline group and borderline regression) and provided evidence of consistency across the different methods [6].

**Fig. 1:**
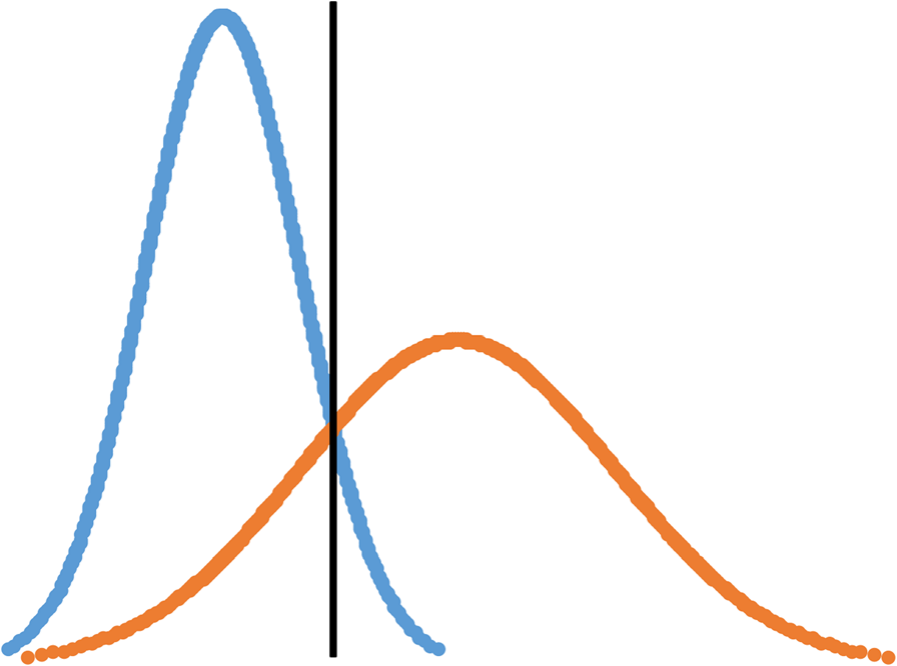
Illustration of the contrasting groups’ method. Blue represents the novice group. Orange represents the experienced group. The black vertical line goes through the identified intercept of the curves, representing the pass/fail cut-off score

When using the contrasting groups’ method, the cut-off point is set by identifying the intercept of normally distributed curves that represent the score distributions of the groups defined by their level of expertise (Fig. 1). Since many validity studies already include groups defined by expertise level, contrasting groups can be considered an easy and feasible method for standard setting in many validity studies. After a pass/fail score is defined, percentage of false positives and false negatives can be calculated to explore the consequences of the test.

If we consider a study with two groups defined by different levels of expertise, e.g., novices and experts; the false positives are defined as novices who score higher than the pass/fail score and pass the test, and the false negatives are defined as experts who score lower than the pass/fail score and fail the test. Traditionally, these false positives and false negatives are calculated based on the observed number of individuals who passes or fails a test.

**Fig. 2:**
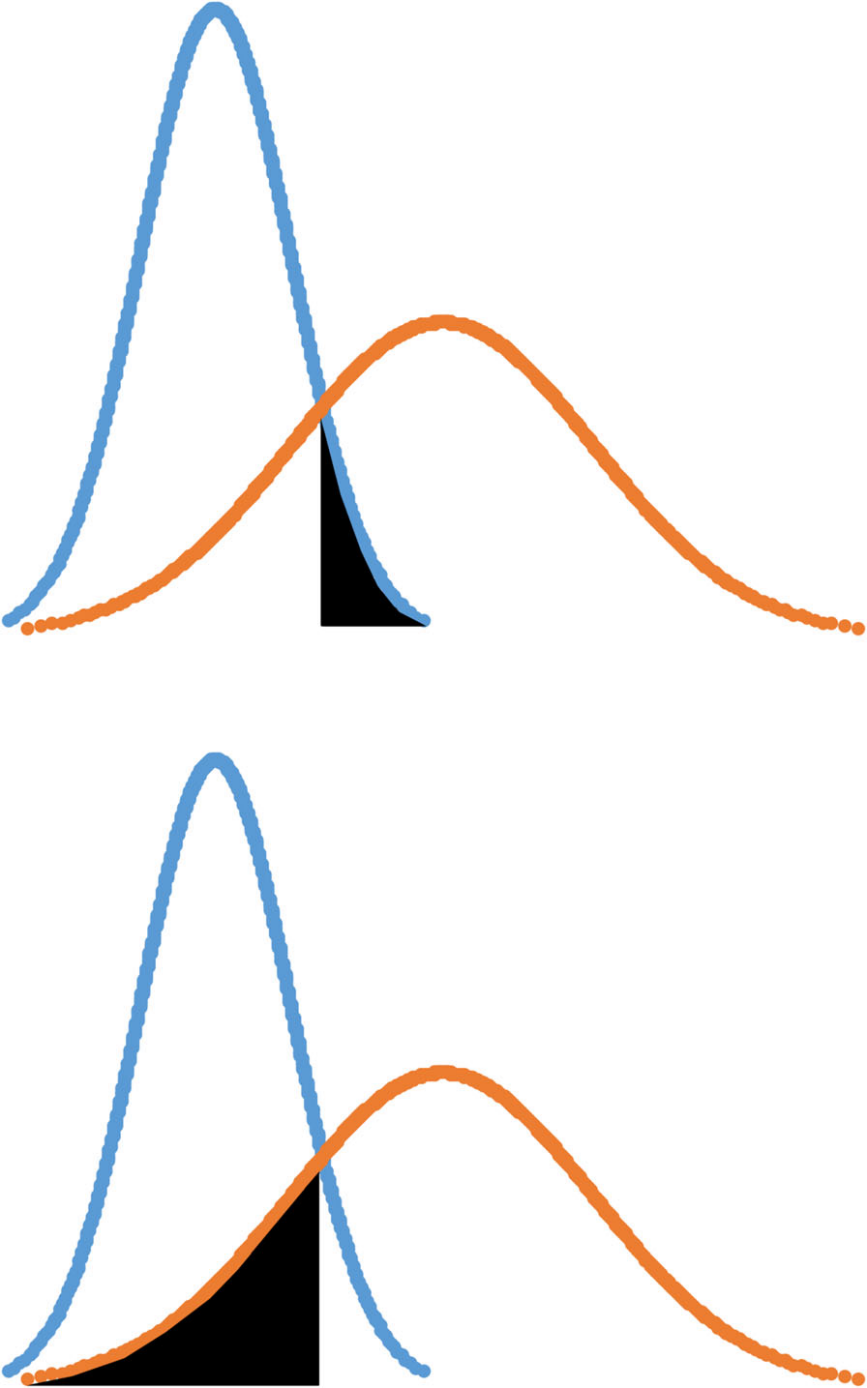
Illustration of the theoretical false positive and theoretical false negative using the normal distribution curve of group-specific scores. Top, the black area represents the cumulative distribution corresponding to the false positives. This is defined as the area under the curve of the blue curve that is to the right of the intercept point of the curves corresponding to the pass/fail cut-off score. Bottom, the black area represents the cumulative distribution corresponding to the false negatives. This is defined as the area under the curve of the orange curve that is to the left of the intercept point of the curves corresponding to the pass/fail cut-off score

Validity studies often include only a small number of participants, especially in the expert group due to limited number of available experts [8]. When performing consequences analysis, these small numbers make the rate of false positives and false negatives particularly sensitive to outliers of the normal distribution curve, which may lead to unrepresentative percentages of false negatives and false positives. Observed false negatives and false positives are calculated using the actual numbers of experts who failed the test and novices who passed the test. This is different from what we call the theoretical false negatives and theoretical false positives, which can be calculated using the normally distributed curves that represent the score distributions of the groups defined by their level of expertise (Fig. 2). While in theory, these observed and theoretical false negatives and false positives should lead to the same results, in practice, they may differ especially when studies are made on small group sizes.

In this paper, we discuss reporting considerations when using contrasting groups for standard setting. We demonstrate that the small number of participants in validity studies make consequential validity analyses very sensitive to outliers, which is a phenomenon with important implications for how we collect and interpret validity evidence. We propose that theoretical false positives and false negatives should be reported in addition to observed false positives and false negatives. Finally, to facilitate consequential validity analyses in future validity studies, we have developed an Excel sheet which can determine the pass/fail score when group-specific descriptive statistics are given (mean, standard deviation, group size) (Additional file 1). The Excel sheet can also calculate the theoretical false positives and theoretical false negatives.

## Methods

In a recent systematic review, Goldenberg et al. identified studies for establishing absolute standards for technical performance in surgery [9]. We independently extracted data from studies identified by Goldenberg et al., which used contrasting groups’ standard setting for a consequences analysis. We calculated the observed false positives and false negatives where published data were available on the pass/fail cut-off score, the mean and standard deviation of each group, and the number of participants passing and failing in each group. For each group, we also calculated the theoretical false positive and theoretical false negative using the cumulative distribution function. The cumulative distribution of a real-valued random variable *X* where the probability of *X* being less than the value *x* can be described as the following:

$$ f(X)=P\left(X<x\right) $$

We constructed a score distribution of the novices using the extracted mean and standard deviation, and used the probability of the random variable *X* being more than the pass/fail cut-off score to calculate the theoretical false positives (Fig. 2). We constructed a score distribution of the experts using the extracted mean and standard deviation, and used the probability of the random variable *X* being less than the pass/fail cut-off score to calculate the theoretical false negatives (Fig. 2).

To ease the conduct and reporting of contrasting groups analyses, we developed an Excel sheet which is available as a supplementary material to this paper (Additional file 1). Using group-specific mean, standard deviation, and number of participants, the Excel sheet automatically calculates a pass/fail cut-off as well as theoretical false positives and theoretical false negatives. The Excel sheet is also used for the examples provided in the results.

## Results

None of the studies examined reported theoretical false positives and theoretical false negatives as defined in the present paper [6, 10–26]. The following interesting examples illustrate how small groups in validity studies make the observed false positives and observed false negatives sensitive to the outliers of the distribution curve.

**Fig. 3:**
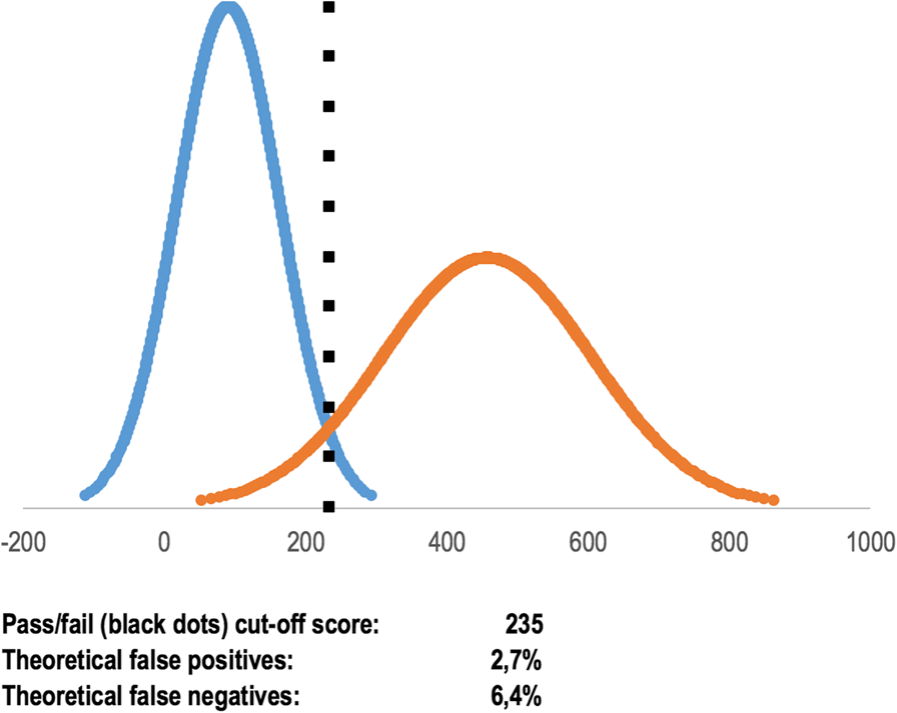
Example of contrasting groups’ method with data on theoretical false positives and theoretical false negatives. In this case, the authors of the study observed that one novice passed (observed false positive rate of 9.1%) and that no experts failed (observed false negative rate of 0.0%). The theoretical false positives and theoretical false negatives suggest that if the groups had been much larger, 2.7% of the novices would have passed the test and 6.4% of the experts would have failed the test

Nerup et al. explored validity of an automated assessment tool on 11 trainees in colonoscopy and 10 experienced endoscopists [17]. The two groups of participants performed colonoscopy in two case scenarios on a realistic standardized model of the human colon. A pass/fail score was established by using the contrasting groups’ method. In one of the cases explored, one trainee had a score higher than the established pass/fail score and passed the test (observed false positive rate 9.1%), whereas no experts failed (observed false negative rate 0.0%). Using the cumulative distribution function, we calculated that theoretically 2.7% of the trainees should have passed the test and 6.4% of the experts should have failed the test (Fig. 3). These numbers correspond to 0.3 participant in the trainee group and 0.6 participant in the expert group, demonstrating an example where the observed false positives/negatives are sensitive to outliers from the normal distribution curve due to the small number of participants. In this case of an automated assessment tool on colonoscopy, the theoretical false positives and false negatives provide important supplementary information on the quality of the test.

**Fig. 4:**
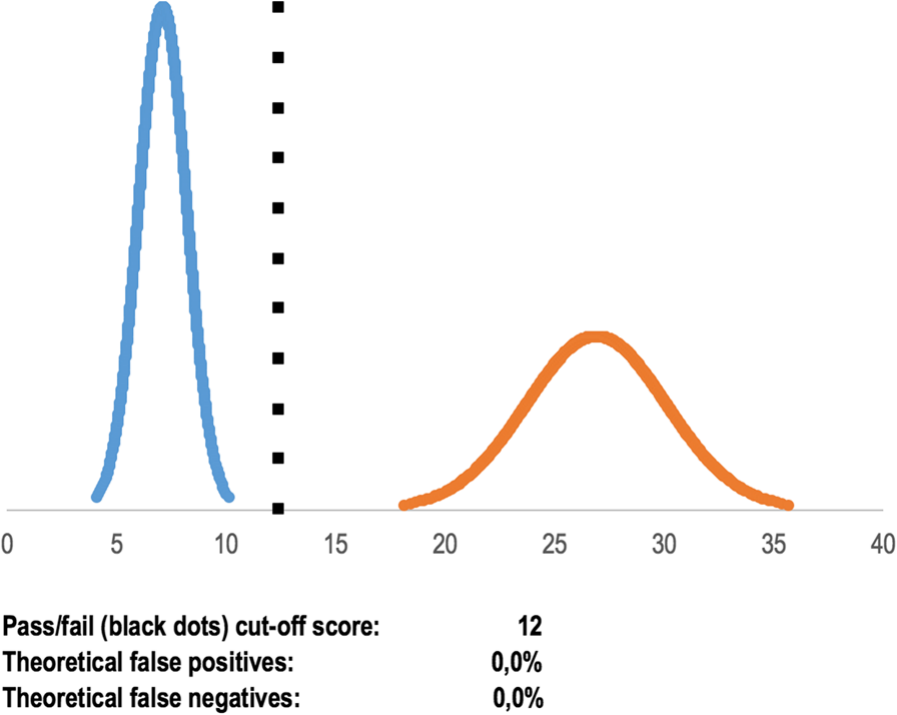
Example of contrasting groups’ method with data on theoretical false positives and theoretical false negatives. In this case, the authors of the study observed that one novice passed (observed false positive rate of 6.7%) and that one expert failed (observed false negative rate of 10.0%). The theoretical false positives and theoretical false negatives suggest that if the groups had been much larger, 0.0% of the novices would have passed the test and 0.0% of the experts would have failed the test

Preisler et al. explored validity of a virtual reality simulator test on 15 trainees and 10 experienced endoscopists [19]. The contrasting groups’ method was used to establish a pass/fail score. One trainee obtained a score higher than the established pass/fail score and passed the test (observed false positive rate 6.7%), whereas one expert failed (observed false negative rate 10.0%). Again, using the cumulative distribution function, we calculated that theoretically 0.0% of the trainees should have passed the test and 0.0% of the experts should have failed the test (Fig. 4). In this case, the outliers of the normal distribution curve significantly affected the observed false positives and false negatives. This would not have been apparent without the information provided by the theoretical false positives and false negatives.

**Fig. 5:**
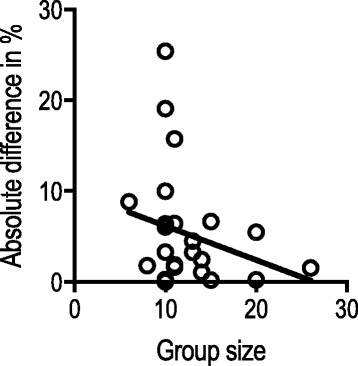
Group size (*x* axis) in relation to the absolute difference between observed and theoretical false positives/negatives (*y* axis). Trend (black line) suggests relationship between group size and precision of the observed false positives/negatives

To illustrate this phenomenon on a larger scale, we extracted pass/fail details from all studies identified by Goldenberg et al. [9] and where it was possible to perform such calculations **(**Table 1**)**. The absolute difference between the observed false positive/negative rate and the theoretical false positive/negative rate is illustrated in relation to the group size (Fig. 5). The figure illustrates that larger group sizes decrease the absolute difference between the observed and theoretical false positives and false negatives and therefore makes the calculations of the observed false positives and false negatives less sensitive to outliers of the distribution curve. This finding suggests that reporting theoretical false positives and negatives are especially important when the group sizes are small.

**Table 1:** All data extracted from studies examined in this paper. In addition, we have calculated theoretical false positives (FP) and theoretical false negatives (FN) and provided the absolute difference between observed and theoretical FP and FN.

Ref.	Number of novices	Novices’ score, mean (SD)	Number of experts	Experts’ score, mean (SD)	Pass/fail cut-off score	Novices passed (observed FP), *n* (%)	Calculated theoretical FP, %	Absolute difference in FP	Experts failed (observed FN), *n* (%)	Calculated theoretical FP, %	Absolute difference in FN
[6]	Data only available for one group										
[10]	20	244 (88)	20	446 (52)	358	2 (10.0%)	9.8%	0.2%	2 (10.0%)	4.5%	*5.5%*
[11]	13	38.6 (27.3)	13	0 (9.1)	15.5	2 (15.4%)	19.9%	4.5%	1 (7.7%)	4.4%	3.3%
[12]^a^	10	1.5 (0.4)	10	4.4 (0.4)	3	0 (0.0%)	0.0%	0.0%	0 (0.0%)	0.0%	0.0%
[12]^b^	10	1.8 (0.2)	10	3.9 (0.5)	2.5	0 (0.0%)	0.0%	0.0%	0 (0.0%)	0.3%	0.3%
[13]	14	0.27 (0.065)	14	0.65 (0.117)	0.42	0 (0.0%)	1.1%	1.1%	0 (0.0%)	2.5%	2.5%
[14]	No numbers on pass/fail										
[15]	No numbers on pass/fail										
[16]	Data only available as median and range										
[17]^c^	11	93.1 (73.4)	10	459.7 (147.5)	235	1 (9.1%)	2.7%	*6.4%*	0 (0.0%)	6.4%	*6.4%*
[17]^d^	11	41.4 (35.5)	10	106.9 (102.5)	93	1 (9.1%)	7.2%	1.9%	7 (70%)	44.6%	*25.4%*
[18]	26	333 (96)	11	497 (52)	422	5 (19.2%)	17.7%	1.5%	1 (9.1%)	7.5%	1.6%
[19]^e^	15	7.2 (1.1)	10	27 (3.2)	15.5	1 (6.7%)	0.0%	*6.7%*	1 (10.0%)	0.0%	*10.0%*
[19] ^f^	15	0.32 (0.31)	10	2.48 (1.09)	0.79	1 (6.7%)	6.5%	0.2%	0 (0.0%)	6.1%	*6.1%*
[20]	10	30 (32)	10	76 (10)	58	0 (0.0%)	19.1%	*19.1%*	1 (10.0%)	3.6%	*6.4%*
[22]^g^	8	0.098 (0.074)	6	0.240 (0.037)	0.19	1 (12.5%)	10.7%	1.8%	0 (0.0%)	8.8%	*8.8%*
[23]	Did not use contrasting groups										
[24]	11	2.7127 (2.25645)	10	0.7890 (0.39156)	1.51	5 (45.5%)	29.7%	*15.8%*	0 (0.0%)	3.3%	3.3%
[25]	No numbers on pass/fail										
[26]	No numbers on pass/fail										

Abbreviations: *SD* standard deviation, *FP* false positives, *FN* false negatives

An absolute difference of > 5% between the observed and theoretical FP and FN are marked in italics

^a^Transabdominal novices

^b^Transvaginal novices

^c^Data from case 1

^d^Data from case 2

^e^Data from the virtual reality model

^f^Data from the physical model

^g^Mean and standard deviation are estimated from median and interquartile range

## Discussion

The contrasting groups’ standard setting method is commonly used for consequences analysis in small-sized validity studies. Here, we demonstrate that the observed false positives and observed false negatives are very sensitive to small samples and outliers. We argue that theoretical false positives and theoretical false negatives should be provided in addition to the observed false positives and observed false negatives. However, it should be noted that the theoretical false positives and theoretical false negatives are based on mean and standard deviation of the same small samples; hence, it cannot solve a problem of small samples in a study, but may provide an important addition to considerations on false positives and false negatives in consequences analyses.

The passing score can be moved from the intersection point between the two normal distributed curves if there is a greater concern of either passing non-competent participants or failing competent participants [7]. When having such considerations, information of both observed and theoretical false positives and false negatives should be considered. These considerations are essential when setting standards and can be used actively to set a pass/fail score that fits with the rate of false positives and false negatives. One limitation of this paper is that we have not described details of cases where it may be relevant to move the passing score from the intersection point to address other needs, e.g., if a certain level of sensitivity or specificity is of interest. Such cases may benefit from receiver operator curve-based analyses [26, 27].

The differences between the observed and the theoretical false positives and false negatives should be reduced as the group samples are increased. In studies with large groups, the value of providing theoretical false positives and false negatives in addition may be limited since the distribution more closely will resemble the normal curve and the differences between observed and theoretical false positives and false negatives would be small. However, providing theoretical false positives and false negatives may still be relevant to underline the conclusions of such studies.

Using the contrasting groups’ method requires two groups defined by clear differences in expertise, i.e., novices who are not supposed to pass the test and experienced individuals who a priori should all pass the test. Collecting a sizeable group of non-competent performers can be a challenge, especially in a clinical environment where obvious ethical reasons do not allow complete novices to practice on patients. For example, in the Montbrun et al. study of technical performance by surgical trainees, the authors were unable to use calculations based on contrasting groups in some cases because of too few individuals in the novice/incompetent group [6]. In such cases, borderline-based methods are more feasible, but this method often requires big sample sizes before ‘enough’ trainees are judged as borderline. One can also argue that identifying groups at the border of passing can be a challenging endeavor, e.g., a judge that is unfamiliar with the technique is more likely to judge a participant as a borderline which introduces an assessor bias [9]. Therefore, using contrasting groups can be more feasible in some cases and especially in simulation-based studies where it is safe and ethically sound to let a novice group perform procedures without supervisor interference. It is important to remember that there are no general rule to which method to use, instead the most appropriate method may differ from one study to another based on the purpose of the individual study [9].

## Conclusion

Based on the considerations made in this paper, we recommend reporting theoretical false positives and theoretical false negatives in addition to the observed false positives and observed false negatives in the consequences analyses of validity studies on standard settings using the contrasting groups’ method. This approach may strengthen the consequences analyses, especially when group sizes are small. To facilitate this, we have developed an Excel sheet to ease the conduction and reporting of contrasting groups analyses, which is available as a supplementary material to this paper (Additional file 1).

## Additional file

Additional file 1: Excel file to ease conduction and reporting of contrasting groups analyses. (XLSX 234 kb)
